# Camera Arrangement Optimization for Workspace Monitoring in Human–Robot Collaboration

**DOI:** 10.3390/s23010295

**Published:** 2022-12-27

**Authors:** Petr Oščádal, Tomáš Kot, Tomáš Spurný, Jiří Suder, Michal Vocetka, Libor Dobeš, Zdenko Bobovský

**Affiliations:** 1 Department of Robotics, Faculty of Mechanical Engineering, VSB-TU Ostrava, 70833 Ostrava, Czech Republic; 2 Moravskoslezský Automobilový Klastr, z.s., Business Incubator VŠB-TU Ostrava, 70833 Ostrava, Czech Republic

**Keywords:** workspace monitoring, camera, human–robot interaction, collaboration, sensors network

## Abstract

Human–robot interaction is becoming an integral part of practice. There is a greater emphasis on safety in workplaces where a robot may bump into a worker. In practice, there are solutions that control the robot based on the potential energy in a collision or a robot re-planning the straight-line trajectory. However, a sensor system must be designed to detect obstacles across the human–robot shared workspace. So far, there is no procedure that engineers can follow in practice to deploy sensors ideally. We come up with the idea of classifying the space as an importance index, which determines what part of the workspace sensors should sense to ensure ideal obstacle sensing. Then, the ideal camera positions can be automatically found according to this classified map. Based on the experiment, the coverage of the important volume by the calculated camera position in the workspace was found to be on average 37% greater compared to a camera placed intuitively by test subjects. Using two cameras at the workplace, the calculated positions were 27% more effective than the subjects’ camera positions. Furthermore, for three cameras, the calculated positions were 13% better than the subjects’ camera positions, with a total coverage of more than 99% of the classified map.

## 1. Introduction

Human–robot collaboration (HRC) is currently one of the most developing areas of research, with applications not only in industrial and service robotics but also in elderly care, rescue robotics, intelligent vehicles and aircraft, rehabilitation technology and space applications [1,2]. HRC combines human capabilities with robot precision and efficiency [3]. Robots that enable collaboration in a shared space with humans are referred to as collaborative robots [4]. Collaborative robot workplaces are designed such that the robot performs the part of the operation that requires high precision or that may be non-ergonomic, repetitive, or even dangerous for humans, while the human does the part of the cycle that requires dexterity, intuition, or unique decision making [5]. Such collaboration cannot be performed without a shared workspace [3].

When sharing a workspace, collisions can occur when individual pieces of hardware collide with each other or with some objects in the environment. If only robots move in the shared workspace, their work cycle can be uniquely determined by the control system and then collisions can be avoided by applying suitable algorithms, such as elastic strips [6], artificial potentials [7], or other similar variants [8,9]. In the case where the robot’s workspace is shared with a human, the system cannot unambiguously define the worker’s motion, but only predict it to a limited extent, and, thus, collisions between the robot and operator may occur during the work cycle [10,11]. In HRC systems, operator safety is the most important criterion [12,13,14]. In order to ensure maximum operator safety, various safety-related requirements are imposed on the collaborative workstation according to standards, see [15,16] for a review. According to [17,18], for example, safety zones are defined in the workspace, in which the magnitudes of robot speeds or actuator torques are adjusted to a safe value.

Despite the fact that it pays to invest in smart factories [19], workplaces with robots still do not sufficiently solve some problems related to sharing workspace with humans. A collision, even a safe one, most often leads to an immediate stop of the robot [14], or other collision resolution strategies are used based on the collected data [20,21]. In all cases, however, this means increased duty cycle time [12], resulting in higher energy consumption and making the operation more expensive. For this reason, it is advantageous to use non-contact systems for collision prediction and avoidance [22,23]. Monitoring the shared space is often based on camera systems to detect humans [24] or any obstacles [25]. One use of these camera systems is in conjunction with human–robot interaction (HRI) systems that, for example, use special gloves [26,27] to inform the operator of a possible collision with a robot. This allows the operator to react to this event and adapt his or her movements to avoid a collision. Another way of avoiding a predicted collision is to re-plan the robot’s trajectory in time according to the collected real-time data [12,28].

A disadvantage of camera systems is the possibility of obscuring their surveillance area [29]. The robot’s movement and the operator’s activity can obscure this space during the operation. This creates a volume in the workspace that is not monitored; thus, it cannot be clearly determined whether or not an obstacle is present, which may lead to a collision. Eliminating or minimising the shaded volume of the workspace can be achieved by increasing the number of cameras and by choosing their appropriate placement [25]. 

The correct position of cameras has a significant impact on the performance of the system, and this position depends on the purpose of the system itself. Although camera positions can have a large impact on the quality of the acquired data, there are currently not many methods for determining the optimal number of cameras and their placement for workspace monitoring. For example, the work in [30] describes a method for determining the positions of cameras to provide the best input information relative to the actions performed by a human (recognize motion, learn activities, take measurements, etc.). The authors in [31] describe a simple 2D algorithm that minimises camera view frusta overlap, while not considering any shadowing caused by obstacles, nor different importance of various sections of the workplace. The paper [32] investigates how to deploy the cameras in such a way that the 3D data error is minimized. First, an analytical uncertainty method based on minimizing the error criterion was used, followed by evolutionary optimization methods similar to genetic algorithms.

There is also a method for placing sensors in 3D space called CamHunt [33], described in the example of using it to place cameras inside rooms and the whole building. CamHunt uses a 3D grid partitioning of the environment for camera placement, where the goal is that each voxel is seen by at least one camera. However, it is not described in considering the usage of the robot and its influence. On the other hand, it uses the methodology of multi-camera system placement focused on human presence [34].

Another method addresses placing two different types of sensors (depth and presence) in a shared workspace [35]. It proposes the placement of sensors according to a probabilistic framework computed based on the presence or depth map of obstacles on their image plane, including the robot manipulator maps.

This paper discusses the classification of the workspace shared between the robot and human. Based on the classified map, the camera covering the most space with respect to the importance of the space is searched. Multiple cameras can be found this way: each additional camera added to the workspace covers the space not covered by the cameras already used. This process also takes into account dynamic objects (e.g., the robot). The main motivation is to achieve the necessary workspace coverage using as few cameras as possible, in order to minimize purchase costs, maintenance costs and energy consumption.

Our hypothesis is that camera(s) arranged by human subjects using intuition will provide less coverage of the workspace compared to camera(s) arranged by the algorithm.

## 2. Space Classification

**Figure 1:**
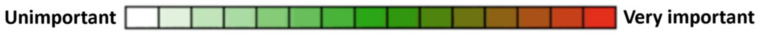
Color coding of space classification importance index ranking from unimportant (white) to very important (red).

In order to design camera positions for workplace monitoring, the workplace must be thoroughly studied. The idea of space classification evaluates parts of the space using an importance index. The importance index describes the impact of the examined volume in the workspace. For clarity, the importance index will be visualised using a colour gradient (see Figure 1), where pure red represents the most important area (an area that is very important for monitoring as there is a very high risk of collision with the robot), green represents the areas with a lower importance (less important for monitoring), and white represents areas, where no collisions can occur (zero importance) and, thus, there is no need for any monitoring. 

The first step is to discretise the workspace into a voxel grid. Each voxel in the grid (a small cube) represents an area with a specific value of the importance index which will be calculated by classification of the space. Dangerous objects, such as the robot, will be integrated into the grid.

To classify the space, classification functions have to be defined. These functions represent the importance around the hazardous elements in the voxel map and calculate the above-mentioned importance indices for all voxels. The functions are defined according to the type of workplace, technology, size, and the level of importance wanted to be ensured in the workplace. The number of classification functions is variable. Only the two most important will be described here: classification based on position of hazardous objects in the voxel map, and classification based on direction of movement of hazardous objects. Based on the type or technology of the workplace, it is also possible to classify, for example, by radiation (heat, light, etc.), maximum energy induced by impact, dangerous tools or manipulated objects (e.g., the possibility of a cut wound caused by sharp sheet metal), etc.

The position classification represents and stores information about accumulated proximity of each voxel from all voxels containing dangerous objects (obstacles) in the voxel map. For a specific voxel with centre point $p_{xyz}$, the position classification value $k_{xyz}^{d}$ can be calculated as

(1) $$k_{xyz}^{d}=\overset{n}{\underset{i=1}{\sum }}\left(L_{max}^{d}-||p_{xyz}-o_{i}||\right)$$

where $\left||p_{xyz}-o_{i}|\right|$ represents the distance between the centre of the voxel under investigation $p_{xyz}$ and the centre of a voxel $o_{i}$ containing a dangerous obstacle, and $L_{max}^{d}\;$represents the chosen threshold value for distance. Voxels with $||p_{xyz}-o_{i}||\geq L_{max}^{d}$ are not included at all, their contribution is considered zero rather than negative as the equation would suggest.

**Figure 2:**
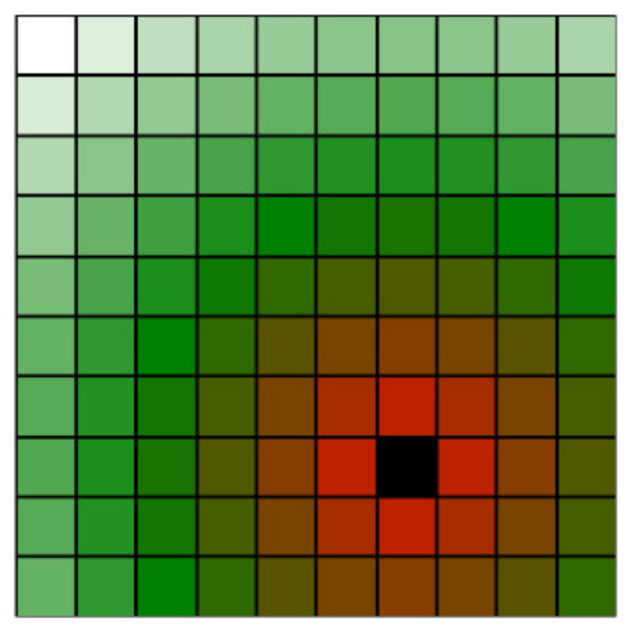
Simplified 2D visualization of position classification of voxels around an obstacle (the black voxel).

This classification creates an imaginary volume around obstacles, whose importance decreases with the distance from the obstacles. Figure 2 demonstrates this principle on a simplified image in 2D space for better clarity. The voxels are represented by squares; the black square contains a dangerous obstacle. Colour coding is according to Figure 1. 

The second classification focuses on the velocity and direction of movement of the hazardous obstacles. It also captures the space around the particles, but voxels in the direction of motion are classified as more important than particles in the opposite direction. For a specific voxel with centre point $p_{xyz}$, the velocity classification value $k_{xyz}^{v}$ can be calculated as

(2) $$k_{xyz}^{v}=\overset{n}{\underset{i=1}{\sum }}\left(\frac{\left||v|\right|}{2}\left(1+\frac{\left(p_{xyz}-o_{i}\right)\cdot v}{\left||p_{xyz}-o_{i}||\cdot ||v|\right|}\right)\cdot \left(\;L_{max}^{v}-||p_{xyz}-o_{i}||\right)\right)\;$$

**Figure 3:**
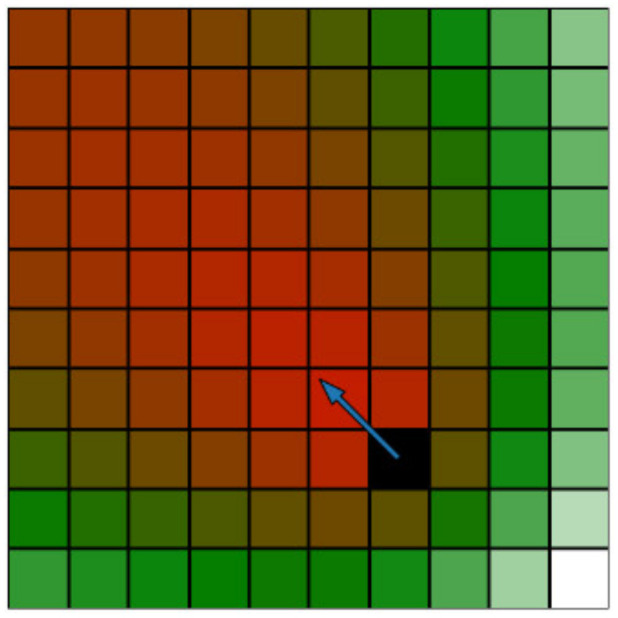
Simplified 2D visualization of velocity classification of voxels around an obstacle (the black voxel); the arrow represents the movement vector of the obstacle.

The impact of the direction of motion is captured by the dot product between the motion vector **v** and the vector pointing from the dangerous voxel $o_{i}$ to the investigated voxel $p_{xyz}$. The dot product is multiplied by a distance factor, decreasing the resulting value with increasing distance from the obstacle. Again, this is performed only for voxels with $||p_{xyz}-o_{i}||<L_{max}^{d}$. A simplified 2D example of velocity classification is show in Figure 3.

It is also important to take into account the access of the worker to the monitored workspace. Typically, the human operator approaches the work area from a single direction or a few directions (e.g., from the front and the left side). This can be simply described by access planes. In the vicinity of these planes, the most frequent occurrence of the operator is assumed and, thus, the importance of collision checking is increased. 

**Figure 4:**
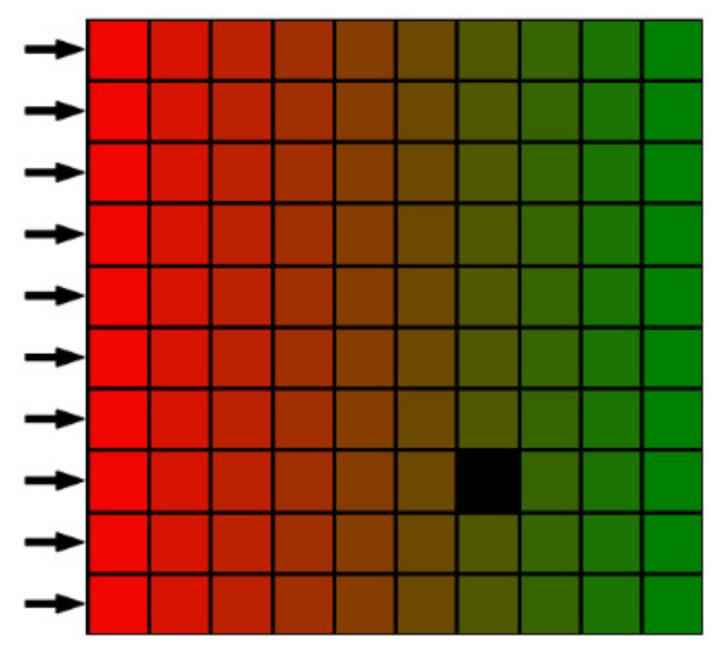
Visualisation of the importance weight in a 2D workspace with one access plane (depicted by the black arrows).

This parameter can be a key feature of classification functions. It determines the importance (weight) of the classification functions by applying a scaling factor $f_{xyz}^{w}$ calculated for a specific voxel and *n* access planes as follows:

(3) $$f_{xyz}^{w}=\overset{n}{\underset{i=1}{\sum }}\frac{L_{max}^{i}-d_{xyz}^{i}}{L_{max}^{i}}\;$$

where $L_{max}^{i}\;$represents the maximum distance from the *i*-th access plane to be considered and $d_{xyz}^{i}\;$represents the distance of the voxel from the *i*-th plane. The importance value $f_{xyz}^{w}$ is equal to one directly at the access plane and decreases linearly until reaching zero at the chosen threshold distance $L_{max}^{i}$. For all voxels with $d_{xyz}^{i}>L_{max}^{i}$, we consider $f_{xyz}^{w}=0$. The visualisation in Figure 4 demonstrates in 2D the weight value corresponding to one access plane (the left side of the image).

The worker access plane is a simple way to include a worker in the classification map. If there are some known predetermined movements that the worker performs in the workplace, the plane can be replaced or supplemented by discrete points or bounding volumes (boxes) in which the worker is frequently located, and weights of adjacent voxels can be affected based on the distances from these points.

The total classification index for a particular voxel is calculated as the sum of the values of all chosen classification functions calculated for this voxel, each multiplied by an optional weight factor $w_{i}$. These additional weights can be introduced to fine-tune the relative importance of individual classification functions, as needed by the specific use-case. The resulting sum is then multiplied by the overall importance scaling factor $f_{xyz}^{w}$ given by the access plane(s). If the number of classification functions is *n*, the total classification index for a given voxel is calculated as follows:

(4) $$k_{xyz}=f_{xyz}^{w}\cdot \overset{n}{\underset{i=1}{\sum }}w_{i}\;\cdot \;k_{xyz}^{i}$$

**Figure 5:**
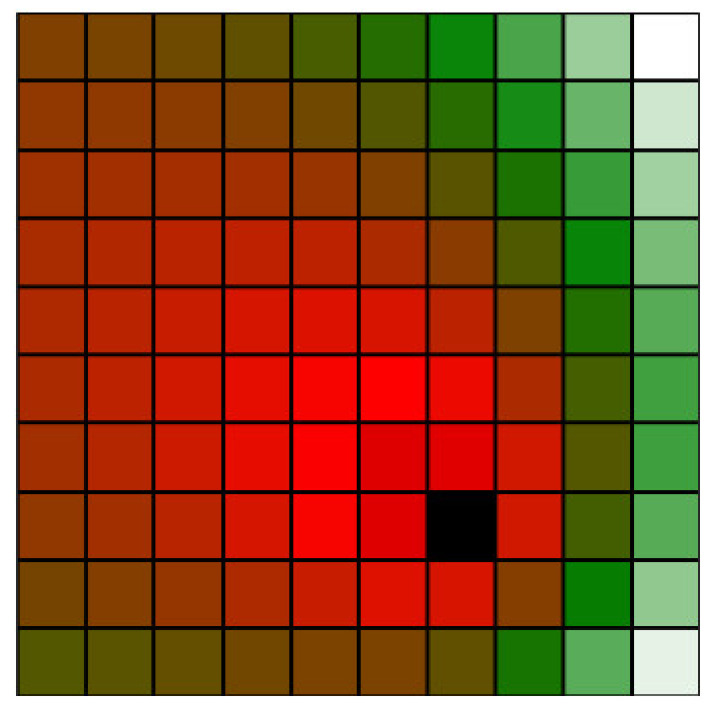
Simplified 2D visualization of the total classification index of voxels around an obstacle (the black voxel).

In our case, we consider two classification functions—see Equations (1) and (2)—and, thus, this equation can be written specifically as follows (the visualisation can be seen in Figure 5):

(5) $$k_{xyz}=f_{xyz}^{w}\;\left(w_{d}k_{xyz}^{d}+w_{v}k_{xyz}^{v}\right)$$

## 3. Camera Classification

Camera classification index is a value that describes how good a camera with a particular position and orientation is in monitoring the workplace. The camera classification *c* is calculated as the sum of classification values $k_{xyz}$ of all voxels visible for the camera (${k^{\prime }}_{xyz})$:

(6) $$c=\overset{n}{\underset{i=0}{\sum }}{k^{\prime }}_{i}$$

**Figure 6:**
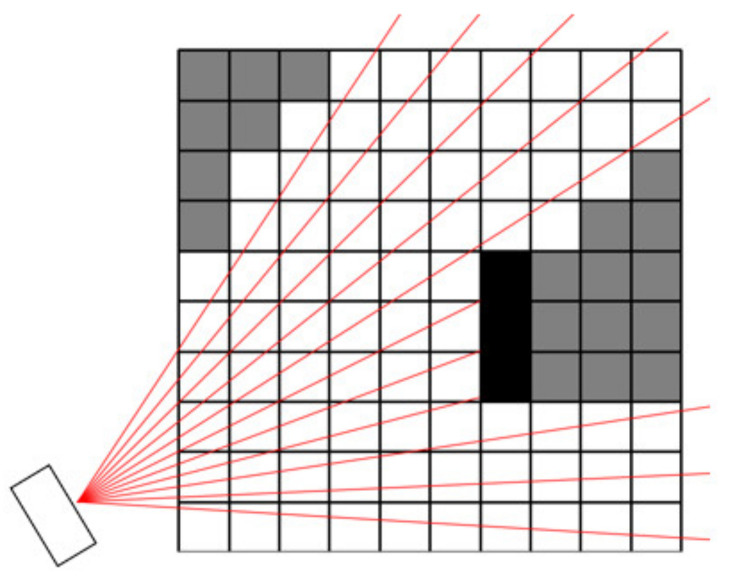
Using camera rays (red lines) to detect visible voxels in the grid; white voxels are visible, grey voxels are invisible, black voxels represent an obstacle.

To find the voxels that are visible to the camera, we cast a ray from the camera origin through each individual pixel of the camera image. Every voxel intersected by some ray is considered visible; see an example in Figure 6, where visible voxels are drawn as white squares and invisible as grey squares. Grey squares represent parts of the workplace that cannot be monitored by the camera. If there is an obstacle (e.g., the robot) in the workspace, the voxels containing the obstacle (black squares in Figure 6) block the rays and, thus, the voxels behind the obstacle are also considered invisible.

The camera classification index (6) represents the coverage of the grid by the camera with respect to the importance of the space. If two cameras capture the same number of voxels, their classification indices may vary according to the importance of the voxels captured.

**Figure 7:**
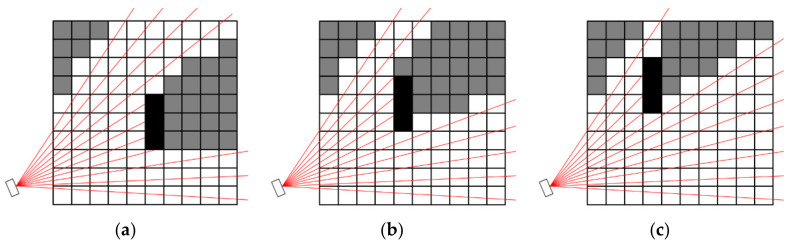
Camera coverage of a voxel map with a moving obstacle (black voxels) during three time moments; (**a**) t_1_; (**b**) t_2_; (**c**) t_3_.

Since the obstacles can move in the environment during the work cycle (typically a robot arm following a trajectory in a cycle), the camera coverage varies during time; see an example in Figure 7.

In this case, the total coverage is calculated as the sum of the camera coverage *c* at each time step during the whole movement cycle from *t* = 0 to *t* = *T*. The percentage of camera coverage over time ($c_{rel}$) is the sum of the coverage $c\left(t\right)$ at each time step divided by the sum of all voxel classification values (including voxels not visible to the camera) during the whole cycle period:

(7) $$c_{rel}=\frac{\sum_{t=0}^{T}c\left(t\right)\;}{\sum_{t=0}^{T}\sum_{i=0}^{n}k_{i}\left(t\right)\;}\cdot 100\;\;\;\left[\%\right]$$

## 4. Finding the Optimal Camera Location

To find the best camera placement for workspace monitoring, we can use Equation (7) to evaluate the cameras and pick the one with the highest value. This can be undertaken either by an optimization algorithm, or by simply trying all possible camera locations (in discrete distances, to obtain a finite number of possible cameras). 

There are six values to optimize: three coordinates for camera position and three values for camera orientation in 3D space. To speed up the optimization process, it is possible to limit the number of variables to three if we determine the camera orientation explicitly by some function based on the camera location. 

**Figure 8:**
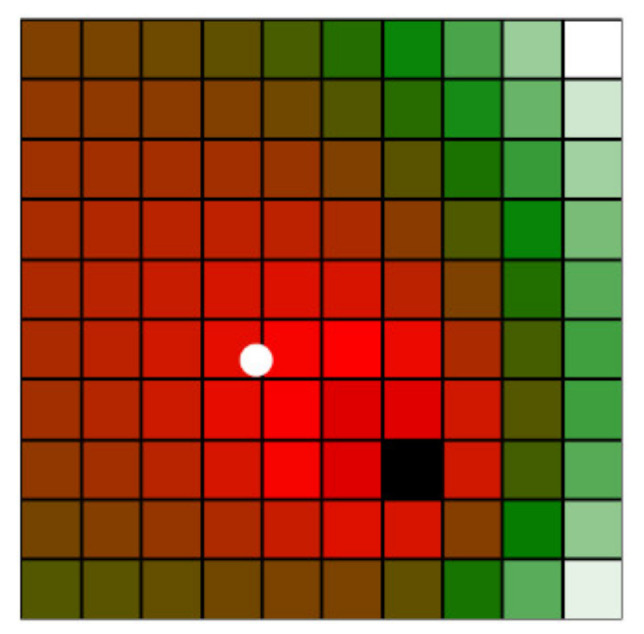
Example of a center of gravity (drawn as a white dot) of a simplified 2D voxel grid.

The camera orientation significantly influences the classification; if the orientation is inappropriately chosen (for example, away from the direction of the voxel grid), the camera may not sense the space at all. To ensure maximum camera coverage, each camera can be focused on the imaginary centre of gravity of the workspace voxel map (the camera axis passes through the centre of gravity). The centre of gravity of the voxel grid is calculated as the centre of gravity of a system of mass points, where the points are defined as voxel centres, and the classification function value represents their mass. Figure 8 shows the centre of gravity for the simplified example.

If multiple cameras are to be used to cover the space, the best camera (with the highest coverage $c_{rel}$) is evaluated first, and then all voxels covered by this camera are devalued by setting their classification index $k_{xyz}$ to zero (separately for each time step during the work cycle *T*). Then, a new grid centre of gravity is computed for the remaining voxels (not covered by the previous camera), and the best camera for these now conditions is found again. This way, a new camera is selected, which serves as a complementary camera for the previous one.

**Figure 9:**
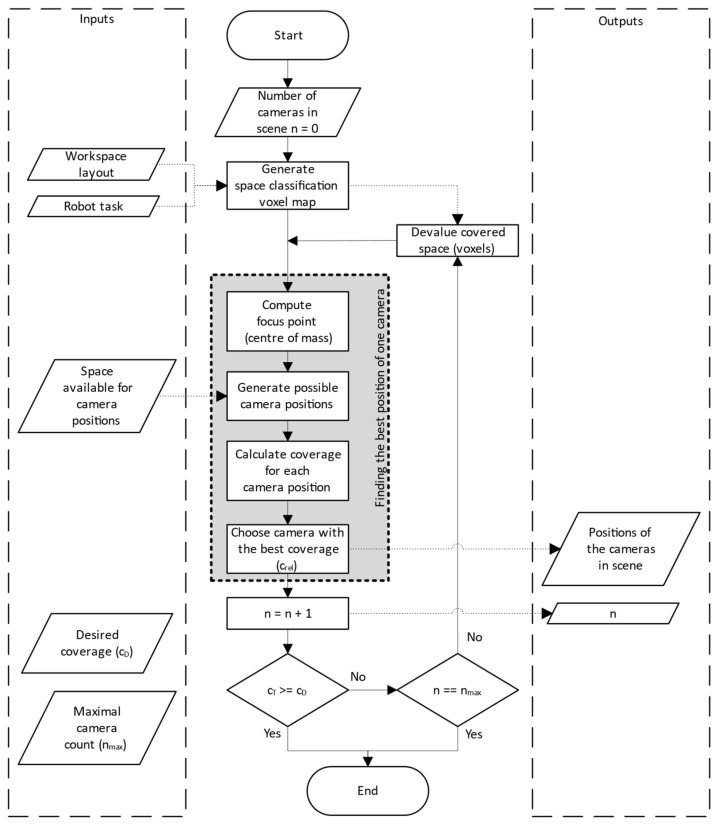
Flowchart describing the whole algorithm.

The whole algorithm of designing a camera subsystem is summarized by the flowchart in Figure 9. The process starts by generating the space classification voxel map according to Section 2, where each voxel has an importance index value that describes its importance for monitoring. This first step depends on the description of the workspace layout, the robot task and on the chosen definition of classification functions; see Equations (1)–(5).

Then, the centre of gravity (focus point) is calculated for the voxel map. The positions of possible cameras are then generated in the defined available space, each camera is oriented towards the focus point. For all these potential camera positions, the camera classification is calculated according to Section 3, which gives each camera its relative coverage; see Equations (6) and (7). The position with the largest coverage is then selected as the best position for that iteration and this camera is added to the list of proposed cameras.

If the total coverage ($c_{T}$) achieved by all proposed cameras (or the single camera, if we are in the first iteration) is sufficient, the process is terminated. If more coverage is needed, it is checked whether the maximum possible number of cameras has not been reached already (the limit must be specified by the user). If this limit has been reached, the process ends, since the required coverage could not be achieved. If the limit has not been exceeded, all voxels visible from the recently added camera are effectively removed from the classification map by setting their importance value to zero and the next iteration of the process is started to find another complementary camera to the already calculated cameras. The result is then a list of camera positions and orientations.

## 5. Experiment

**Figure 10:**
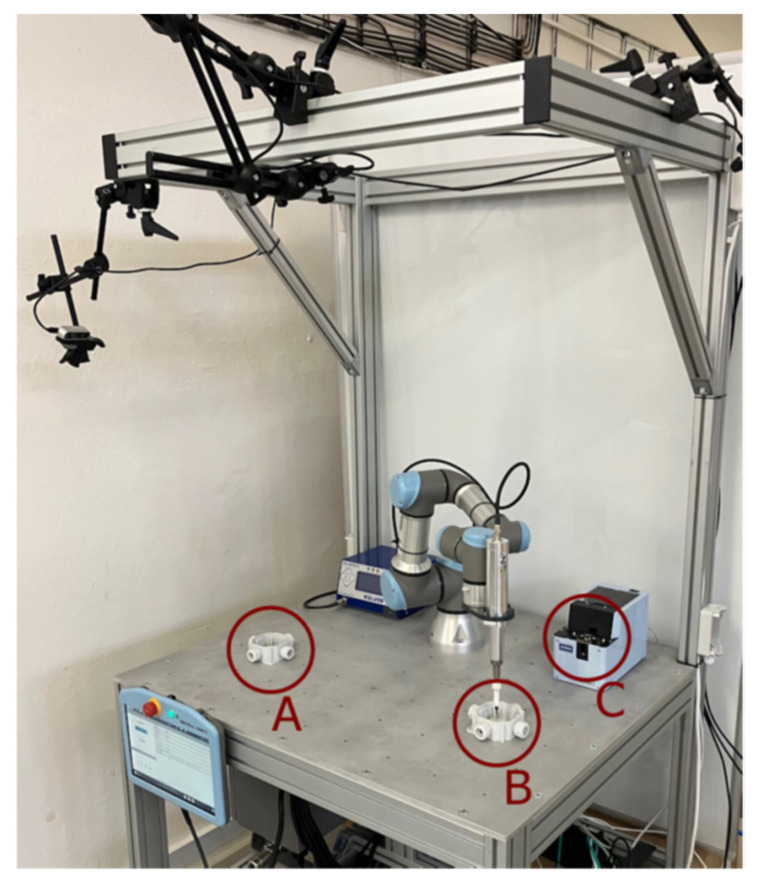
Experimental workplace with the UR3 robot; letters A and B denote the assembly stations, letter C denotes the supply feeder.

**Figure 11:**
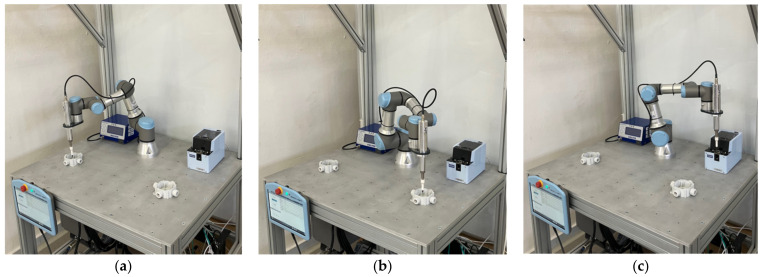
Target positions of the UR3 manipulator during the work cycle: (**a**) assembly station A; (**b**) assembly station B; (**c**) supply feeder at station C.

An experiment was designed to demonstrate the effectiveness of our system. The experiment involves a workstation with the UR3 robot performing an assembly task; see Figure 10. The robot is responsible for picking up a screw from the feeder at station C and screwing it at assembly stations A and B, one at a time (the sequence is C-A-C-B, repeated in a cycle). Meanwhile, the human operator replaces the parts at stations A and B; the operator changes the part at station A while the robot operates at station B and vice versa. The positions of the screwdriver tool in the robot arm in each station are shown in Figure 11.

**Figure 12:**
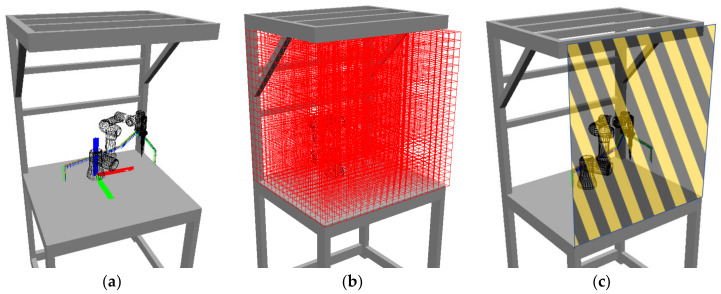
Simulation of the experimental workplace: (**a**) robot trajectory between stations A, B, and C; (**b**) voxel grid covering the workspace volume (voxel size is 5 cm); (**c**) operator access plane to the workplace.

The robot trajectory for the task cycle is visualised in Figure 12a. Visualisation of the overall workspace volume is shown in Figure 12b in the form of a voxel grid with a grid size of 5 cm. Figure 12c shows the operator access plane.

**Figure 13:**
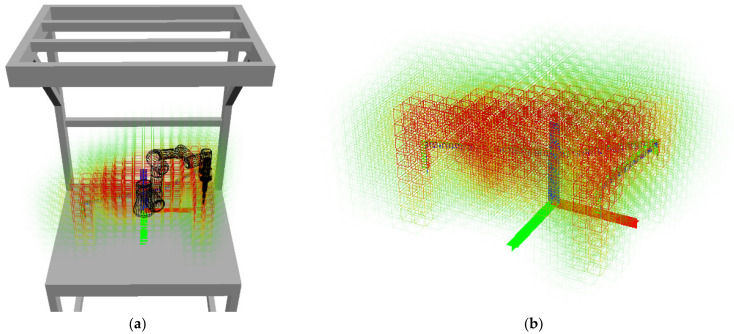
Visualization of the total classification index of voxels in the workplace: (**a**) overall view; (**b**) detail (the coordinate system represents the location of the robot base).

The classification Function (5) was used to calculate the importance of all voxels in the grid. The resulting classified map is shown in Figure 13, using the colour coding from Figure 1. Voxels with zero importance are shown as white, or fully transparent; these voxels do not have to be monitored at all, it is safe for the operator to move in these areas.

**Figure 14:**
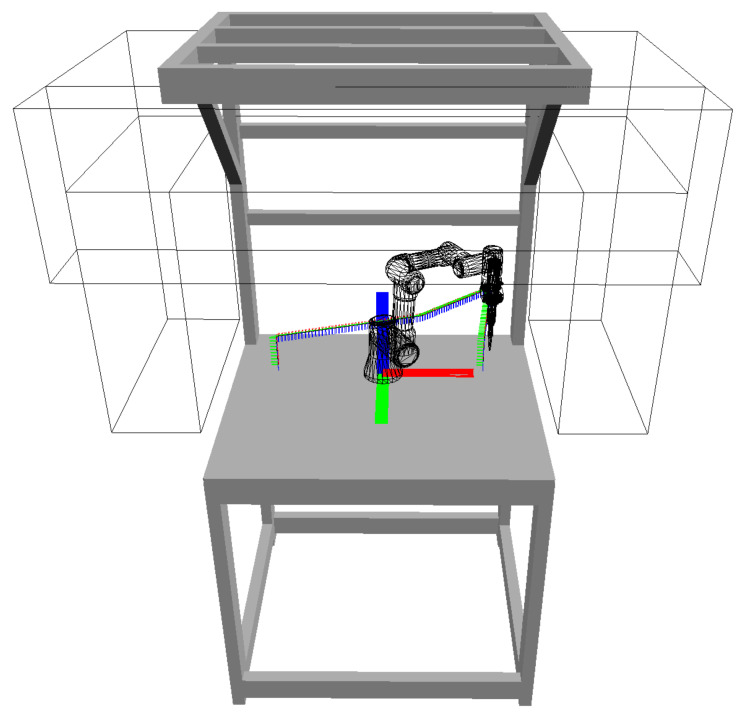
Visualization of the space available for camera placement (gray bounding boxes).

In real applications, there are always some limitations on possible camera locations, given by the presence of some other equipment, human worker movements, available mounting supports for the cameras and their connection, etc. For our experimental workplace, these limitations are fulfilled by placing the cameras only inside any of the bounding boxes visualized in Figure 14.

**Figure 15:**
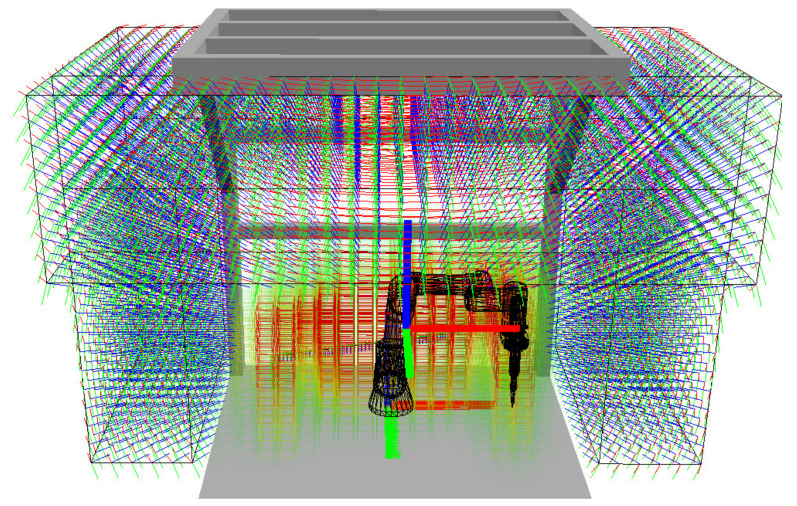
Position and orientation of all possible cameras monitoring the workplace.

All possible locations for cameras are generated inside these bounding boxes in a grid with a chosen spacing of 5 cm in axes *x*, *y* and *z*. The resulting positions and orientations of all possible cameras are shown using the small coordinate systems in Figure 15; the blue axes represent the view directions of cameras towards the centre of gravity of the classified voxel map (represented by the large coordinate system).

The following values of coefficients and weights were chosen for the calculations:

- $L_{max}^{d}$ = 0.2 m (distance threshold for the position classification);
- $L_{max}^{w}$ = 0.4 m (distance threshold for the velocity classification);
- $L_{max}^{1}$ = 0.7 m (distance threshold for the first and only access plane);
- $w_{d}$ = $w_{v}$ = 1 (weights for the two classification functions).

The cameras used in the experiment were Intel Realsense D435, with 1280 × 720 pixels RGB stream, 640 × 480 pixels depth stream, and field of view 87° × 58°.

The camera classification algorithm was used to find the best single camera. Then, the second camera (complementary to the first one) was found. Finally, the third camera, complementary to the other two cameras, was found. Each added camera tries to cover the workspace voxels not covered by the already existing camera(s). In other words, the position of camera number 1 is the same for the case of one, two, and three cameras in the workplace, and the position of camera number 2 is the same for the cases of two and three cameras. 

To verify the benefit of the proposed algorithm, a comparison was made between the quality of workspace coverage using cameras deployed automatically, and cameras placed intuitively a human. In order to obtain statistically relevant data, 30 people were contacted who were completely familiar with the workplace and its specific task. This test group consisted of people with various levels of expertise in the field of robotics and sensory systems, but all of them had a technical education. 

The subjects were given the goal of adjusting the camera positions and orientations to ensure the best possible sensing of the workspace in order to allow the control system of the robot to avoid collisions with the human operator by replanning its trajectory. Every expert placed one camera, then two cameras, and finally three cameras. Each time, they could re-arrange all of them, unlike what the algorithm does with keeping the first (and second) camera in place. The subjects could watch a screen with real-time images from the cameras, to be able to properly evaluate their field of view. However, they did not have access to the calculated workspace classification map; that information is used only by the automated process. The positions and orientations (transformation matrix) of the physically placed cameras were always automatically measured using the 3D grid-board scanning method [36,37], thus allowing subsequent data analysis.

## 6. Results

**Figure 16:**
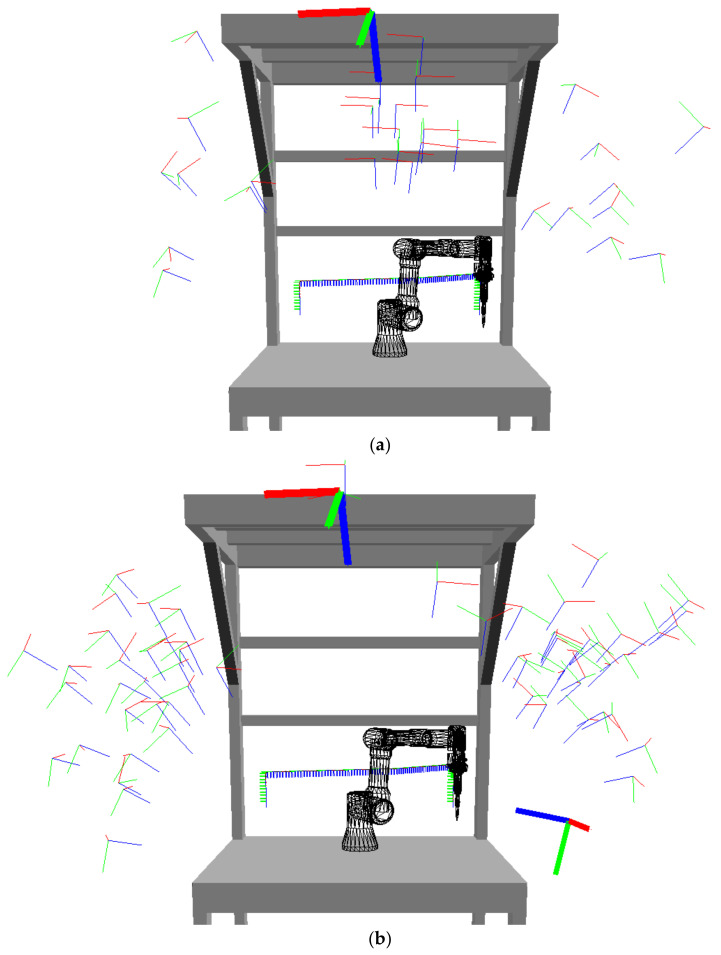

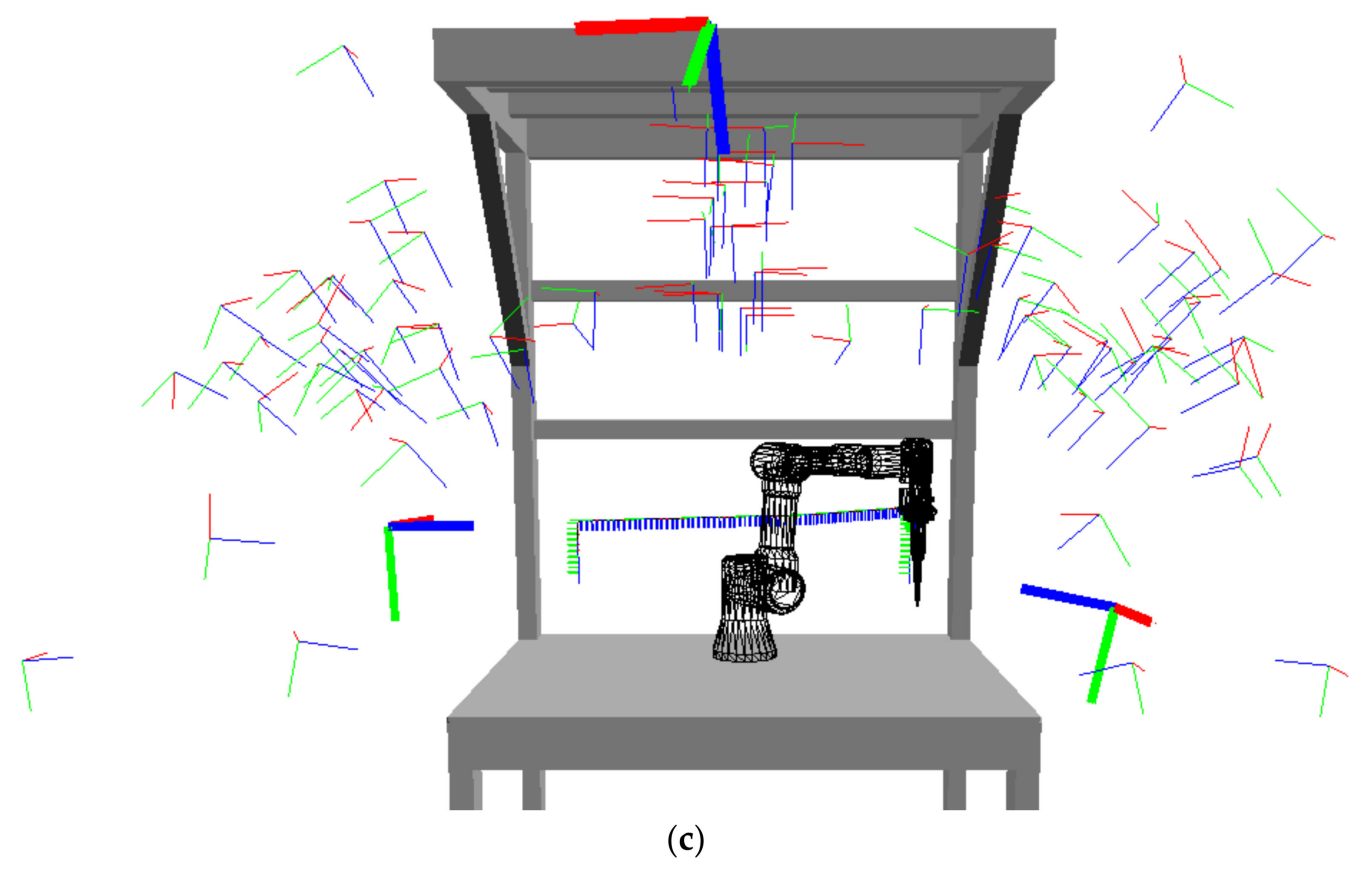
Coordinate systems of proposed camera positions from human subjects (thin lines) and from the automated system (bold lines): (**a**) one camera; (**b**) two cameras; (**c**) three cameras.

Locations of the cameras placed intuitively by human subjects were entered into the simulation environment and the results for the task of placing one, two, and three camera(s) are shown in Figure 16, where the thin lines represent local coordinate systems of all 30, 60, or 90 manually placed cameras (one, two, or three by each technician); and the thick coordinate systems represent the automatically calculated cameras. More than three cameras were not tested, because three cameras were sufficient for the automated system to be able to cover more than 99% of the workspace. As can be seen, even with a small, monitored workplace and a simple robot task, the positions of the cameras vary a lot across test subjects. It is noticeable that most of the cameras are grouped together, but there are also significantly different positions.

**Figure 17:**
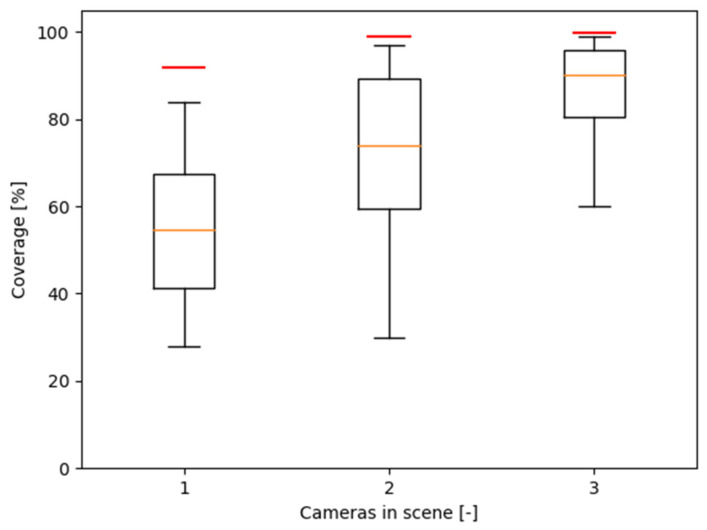
Statistical distribution of relative camera coverage for the camera(s) placed by the human subjects (boxplots), and the relative coverage of the calculated optimal camera(s) (red lines).

The comparison of the effectiveness of the camera positions is based on the percentage coverage $c_{rel}$ of the classified voxel grid. The boxplots in Figure 17 show the distribution of $c_{rel}$ values achieved by human subjects, while the red lines represent the $c_{rel}$ values achieved by the automatically placed camera(s).

As can be seen, with increasing the number of cameras, the subjects were getting closer to the calculation, but the calculated positions were always better. While the average coverage achieved by the subjects was 55.0% for one camera, 72.3% for two cameras, and 87.1% for three cameras, the coverage of the calculated cameras was 91.9% for one camera, 98.8% for two cameras, and 99.8% for three cameras in the workspace. 

**Figure 18:**
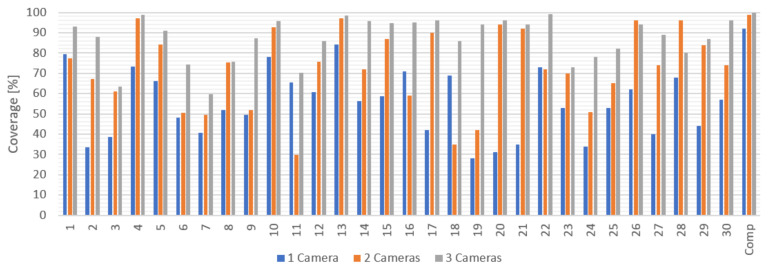
Relative camera coverage achieved by all individual human subjects (1 to 30) and the automated system (“Comp”).

The very best results achieved by the subjects were 84.1% for one camera (subject number 13), 97.2% for two cameras (subject number 4), and 99% for three cameras (subject number 22). Figure 18 shows the achieved relative coverage values for each individual subject and for the automated system.

## 7. Discussion

Since there is no methodology for deploying cameras for workplace monitoring, engineers have to follow their intuition. As shown in the experiment, even for simple trajectories, there are different subjective opinions, and it is not entirely clear where the cameras should be placed. 

The evaluation of one camera in the workspace clearly showed that even if it is a very simple manipulator duty cycle, determining the camera position and orientation is not straightforward for engineers, and the results can vary greatly. Most subjects placed their camera inside an area located in front of the workplace (at the top), and some subjects chose locations on the sides (Figure 16a). Although the locations seem to be quite close together, their workspace coverage differs a lot and the results are not satisfactory. The achieved workspace coverage varies from 28% to 84.1%.

After evaluating two cameras, it can be seen that the maximum subject coverage value is close to the computational system, but the average coverage is well below the computational system coverage. The quality of a pair of cameras varies between human subjects even greatly than for one camera, from 29.8% to 97.2%. Figure 16b demonstrates that all subjects placed one camera on the left side and the second one on the right side.

Even when using three cameras at the workplace, the automated system found the best solution. Dispersion of the coverage values is the smallest here, from 59.7% to 99%. Most subjects placed one camera on the left side, one in the middle, and one on the right side (Figure 16c).

It is worth mentioning that the coverage achieved by the automated system using just a single camera (91.9%) is better than the average coverage achieved by human subjects using two and even three cameras. 

**Figure 19:**
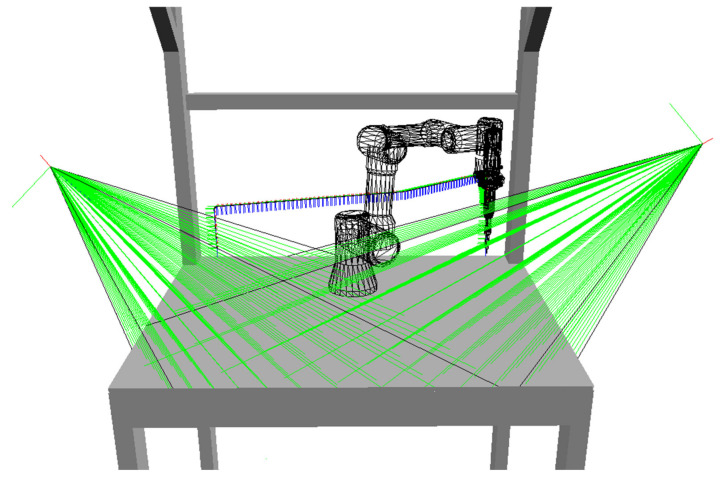
Demonstration of a very bad workplace coverage achieved by subject 11 using two cameras.

Additionally, the cases where a particular subject covered more space with one camera than when using two cameras (subjects 1, 11, 16, 18, and 22, see Figure 18) are interesting. While subject 1 has a slight decrease between one and two cameras, for subject 11, the decrease is very noticeable (more than 50%). In Figure 19, the positions of two cameras proposed by subject 11 are visualised: the cameras are mainly focused down on the workspace table where stations A, B and C are located, while most of the robotic arm is not covered by any camera. This mistake also applied, in some degree, to other subjects and it shows that human intuition can fail in this task. Even though the engineers were supposed to check for collisions of the robotic arm inside the whole workplace, they resorted to capturing mostly the parts with the given technology, instead of the manipulator and its movement.

In general, however, the trend is that the more cameras are used, the more space they cover, even if sometimes only marginally. Even if the subjects have covered the space satisfactorily, there are places in the trajectory that are not covered. These places are also present in the calculated camera positions but are very limited. 

A very good result for the computational system is the fact that with just a single camera, the coverage was better than what 16 subjects (out of 30) achieved with three cameras.

The input parameters of the system, such as the classification functions or the required total coverage ($c_{T}$, see Figure 9), are not universally definable. These parameters are based on the nature of the workplace, and the level of security we want to achieve in the workplace. There are no standards that define these parameters. The effects of these inputs on the placement and number of cameras can be further investigated, but this is not the purpose of this paper.

## 8. Conclusions

The basis of the camera system design in our approach is workspace classification. Classification is used to express the importance index of the space in a discretised voxel grid. The importance index is computed as the sum of the individual classification functions and represents how important it is to monitor the given part of the space (represented by a voxel in the grid) to be able to ensure safety for a human worker. The classification functions are user-defined, as each workplace may have different hazards. These functions may consider, for example, the position, speed or perhaps the amount of energy caused by hazardous objects, typically the robot. The output is then a classification voxel map describing the workspace that needs to be sensed. 

The classification voxel map serves as input for designing camera positions and orientations. Since in most workplaces there are constraints that determine the boundary conditions where the camera can be placed (e.g., it is not possible to place the camera where it would interfere with the operator or other technology, etc.), camera positions are generated only in defined areas of the workplace. In order to define the camera orientation, the centre of gravity of the classified voxel map (where the classification index determines the weight of the position) is computed to determine the camera focus point. 

For the generated camera positions with an orientation towards the centre of gravity of the classified voxel map, the camera indices are calculated to represent the coverage of the classified voxel map (the sum of the classification indices or the voxels that are visible for the camera). The camera with the largest index covers the most space with respect to the space importance indices. 

This whole methodology was verified in a real experiment, where engineers familiar with the workplace task were asked to design a camera subsystem to provide security in the workplace. Subjects were tasked to place one, two, and then three cameras to monitor the workplace. These results were compared with the calculated camera positions. The results confirmed an expected trend that more space is covered when more cameras are used. However, the calculated cameras clearly covered more space than the manually placed cameras. While the average coverage achieved by the subjects was 55.0% for one camera, 72% for two cameras, and 87% for three cameras, the coverage of the calculated cameras was 91.9% for one camera, 98.8% for two cameras, and 99.8% for three cameras in the workspace. Furthermore, the system managed to achieve a better workplace coverage with just a single camera compared to what 16 out of 30 testing subjects managed with even three cameras. Based on the results, we can confirm the hypothesis that the camera system designed by engineers’ intuition achieves lower space coverage compared to cameras arranged by the mathematical model proposed by us.

The purpose of the paper is to provide a methodology for designing a camera subsystem, that will be able to monitor the important sections of a shared workplace in order to prevent collisions between a human worker and a collaborative robot. The importance is, thus, influenced primarily by proximity to the robot arm (the proposed cameras will be able to monitor the robot and its surroundings). The paper does not solve the question how to actually detect the human worker in the monitored workspace using the cameras, that is out of the scope of this research. 

Future work can focus, for example, on the impact of various classification functions and the recommended choice of weights. Another topic worth investigating is the application of optimisation algorithms or neural networks to find the best camera position and orientation instead of the brute-force grid method. With an optimisation algorithm or a neural network, the calculation could be faster, more parameters could be found than just the camera location, and camera positions could be found more precisely than what the grid discretisation is capable of. Furthermore, multiple cameras could be searched together rather than finding one camera and then a complementary second camera.

## Data Availability

There are no data available to share.
